# Maternal PM_2.5_ exposure triggers preterm birth: a cross-sectional study in Wuhan, China

**DOI:** 10.1186/s41256-020-00144-5

**Published:** 2020-05-01

**Authors:** Xiaotong Zhang, Cuifang Fan, Zhan Ren, Huan Feng, Shanshan Zuo, Jiayuan Hao, Jingling Liao, Yuliang Zou, Lu Ma

**Affiliations:** 1 Department of Epidemiology and Health Statistics, School of Health Sciences, Wuhan University, Wuhan, 430071 China; 2 Department of Obstetrics, Renmin Hospital of Wuhan University, Wuhan, 430060 China; 3 Department of Public Health, Wuhan University of Science and Technology School of Medicine, Wuhan, 430081 China; 4 Global Health Institute, Wuhan University, Wuhan, 430071 China

**Keywords:** Preterm birth, Very preterm birth, Moderate preterm birth, Air pollution, LUR model, PM_2.5_

## Abstract

**Background:**

Most of the studies regarding air pollution and preterm birth (PTB) in highly polluted areas have estimated the exposure level based on fixed-site monitoring. However, exposure assessment methods relying on monitors have the potential to cause exposure misclassification due to a lack of spatial variation. In this study, we utilized a land use regression (LUR) model to assess individual exposure, and explored the association between PM_2.5_ exposure during each time window and the risk of preterm birth in Wuhan city, China.

**Methods:**

Information on 2101 singleton births, which were ≥ 20 weeks of gestation and born between November 1, 2013 and May 31, 2014; between January 1, 2015 and August 31, 2015, was obtained from the Obstetrics Department in one 3A hospital in Wuhan. Air quality index (AQI) data were accessed from the Wuhan Environmental Protection Bureau website. Individual exposure during pregnancy was assessed by LUR models and Kriging interpolation. Logistic regression analyses were conducted to determine the association between women exposure to PM_2.5_ and the risk of different subtypes of PTB.

**Results:**

During the study period, the average individual exposure concentration of PM_2.5_ during the entire pregnancy was 84.54 μg/m^3^. A 10 μg/m^3^ increase of PM_2.5_ exposure in the first trimester (*OR*: 1.169; 95% *CI*: 1.077, 1.262), the second trimester (*OR*: 1.056; 95% *CI*: 1.015, 1.097), the third trimester (*OR*: 1.052; 95% *CI*: 1.002, 1.101), and the entire pregnancy (*OR*: 1.263; 95% *CI*: 1.158, 1.368) was significantly associated with an increased risk of PTB. For the PTB subgroup, the hazard of PM_2.5_ exposure during pregnancy was stronger for very preterm births (VPTB) than moderate preterm births (MPTB). The first trimester was the most susceptible exposure window. Moreover, women who had less than 9 years of education or who conceived during the cold season tended to be more susceptible to the PM_2.5_ exposure during pregnancy.

**Conclusions:**

Maternal exposure to PM_2.5_ increased the risk of PTB, and this risk was stronger for VPTB than for MPTB, especially during the first trimester.

## Background

Preterm birth (PTB) is defined as a live birth before 37 gestational weeks [1]. The consequences of PTB include not only fetal and neonatal morbidity and mortality but also potentially lifelong morbidity, including neurologic, pulmonary, and circulatory outcomes [2, 3], which collectively places a substantial burden on affected families, as well as on health and social services [4]. Multiple factors have been suggested to be associated with PTB, including multiple pregnancies, infection, chronic diseases, maternal behavior, and socioeconomic characteristics [5]. Furthermore, ambient environmental factors, such as air pollution, may play an important role in PTB [6, 7].

In recent years, numerous studies have reported the effects of long-term or short-term air pollutant exposure on PTB [8, 9], especially the impact of maternal fine particulate matter (PM_2.5_, aerodynamic diameter < 2.5 μm) exposure on PTB, which has become an intriguing research topic [10–12]. Due to its specific characteristics, including large surface area, small diameter, and extended suspension time in the air [13], PM_2.5_ can be inhaled into the deep regions of the lungs. Oxidative stress and inflammation may be one mechanistic pathway through which exposure to PM_2.5_ triggers the onset of preterm labor [14]. A previous study found that different subtypes of PTB, defined by gestational age, have been associated with different risk factors, including air pollution [15]. Therefore, it is necessary to divide PTB into three categories and examine the relationships between prenatal PM_2.5_ exposure and PTB subtypes. However, very few studies have investigated the association between air pollution and PTB subtypes [16]. Additionally, although researchers have explored the trimester-specific association between PM_2.5_ exposure and PTB [11, 12, 17], the results regarding the most susceptible exposure window have been inconsistent and remain controversial [18]. The inconsistent results among these studies may be attributed to many factors. Aside from the heterogeneity of study areas and populations, the different methods of exposure assessment are also an important reason for the estimate bias, which cannot be ignored [19].

To date, most PM_2.5_-PTB studies in China have based on air pollution data from fixed monitors for the exposure assessment; however, this method is limited in spatial representativeness and might cause exposure misclassification. Interpolated air pollution data from a land use regression (LUR) model can address this weakness by considering additional factors, such as land use, emission, traffic, and population. Although a LUR model has been adopted in some previous PM_2.5_-PTB studies, most of them were conducted in developed countries with low pollution levels [9, 11]. The health effect of particulate matters varies by areas [20], which may be attributed to the differences in chemical composition and population characteristics [21]. Considering the substantial disparity that exists between China and developed countries (for example, chemical compositions of PM_2.5_ and characteristic of population), the results from previous studies regarding air pollution and PTB in developed countries cannot be extended to areas with higher PM_2.5_ concentrations. Therefore, we aimed to use a LUR model to assess individual exposure, and further explore the association between trimester-specific exposure to PM_2.5_ and the risk of premature birth in Wuhan, China.

## Methods

### Study population

**Fig. 1:**
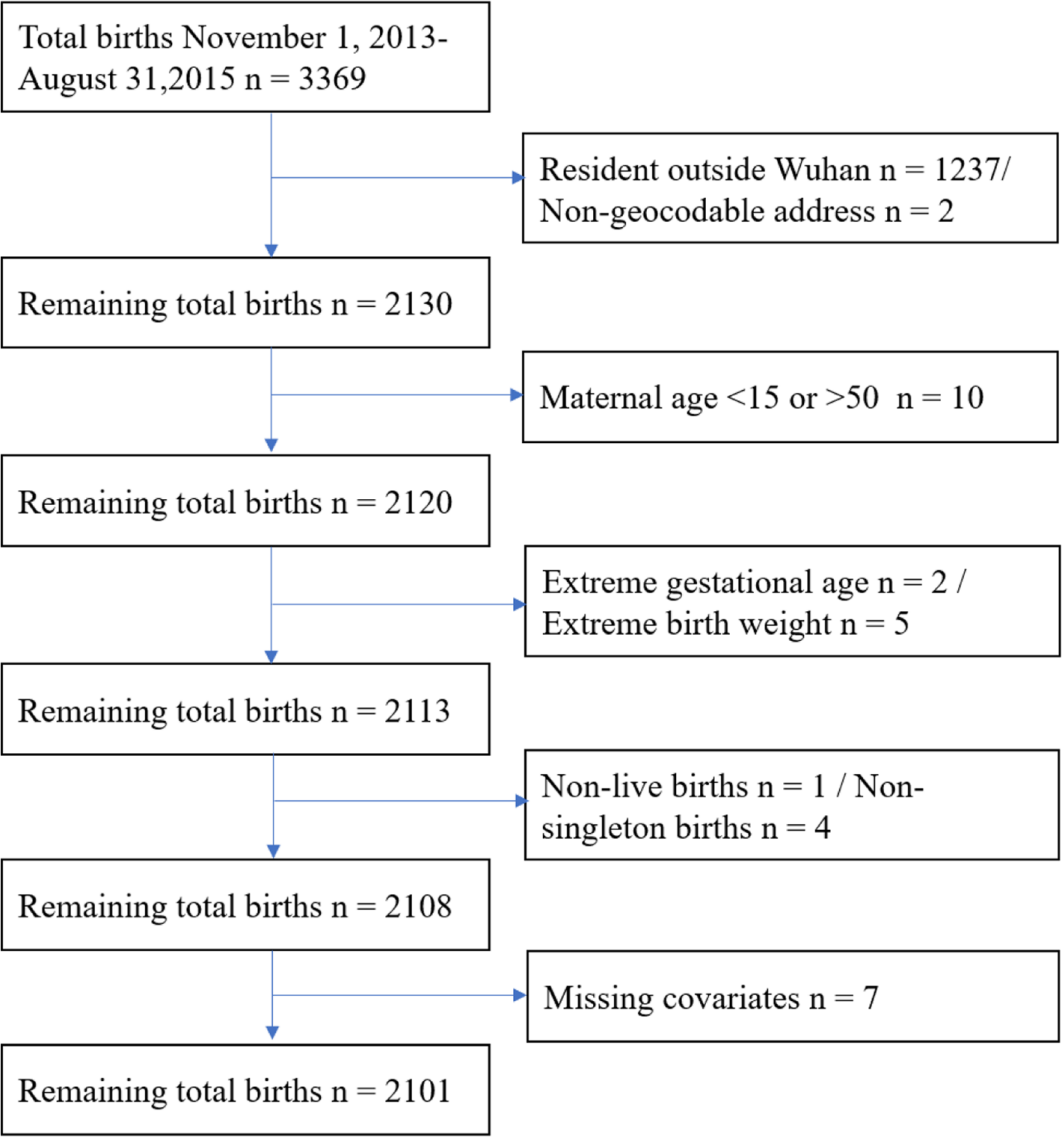
Flow chart illustrating the selection process for the cohort study population

Women who gave birth at one of the 3A hospitals in Wuhan between November 1, 2013 and May 31, 2014; between January 1, 2015 and August 31, 2015 were retrospectively enrolled in this study. Birth records and maternal information used in this study were obtained from the Obstetrics Department in this 3A hospital. This hospital is one of the best hospitals in Hubei province, it has the most advanced medical technology and better health care. So, women in poor physical conditions prefer to deliver at this hospital. Based on inclusion and exclusion criteria in previous researches [12, 22], we excluded multiple pregnancies, stillbirths, birth defects, neonates with an extreme birth weight (< 500 g or > 5000 g), and neonates whose gestational age were less than 20 weeks or more than 42 weeks. Pregnant women whose permanent addresses were not located in Wuhan and whose addresses could not be geocoded were also excluded. To protect the privacy of the individuals, this study only marked the locations of the subjects on the map, without showing accurate longitude and latitude on the map. Finally, a total of 2101 births that met the inclusion criteria were enrolled in this study (Fig. 1).

The covariates were collected from medical records documented by doctors and nurses after the deliveries. The original data were recorded in medical report books and then transformed into electronic form. The collected variables included maternal age, years of education, gravidity, parity, date of birth, gestational age, delivery mode, sex of infant, and maternal physical conditions during pregnancy, including gestational hypertension and gestational diabetes. The date of conception and gestational age were calculated based on the first day of the last menstrual period (LMP), which was recorded on the registration of delivery.

### Definition of birth outcomes and exposure window

The entire pregnancy was defined as the conception date to birth. The first trimester was defined as the conception date to 13 weeks, the second trimester as 14 weeks to 27 weeks, and the third trimester as 28 weeks to birth [16]. Pregnancy outcomes in this study included term birth (gestational age ≥ 37 weeks and ≤ 42 weeks), PTB (20 to < 37 gestational weeks) [1], extremely preterm birth (ExPTB, < 28 weeks), very preterm birth (VPTB, 28 to < 32 weeks), and moderate preterm birth (MPTB, 32 to < 37 weeks) [12].

### Source of air pollutant

**Fig. 2:**
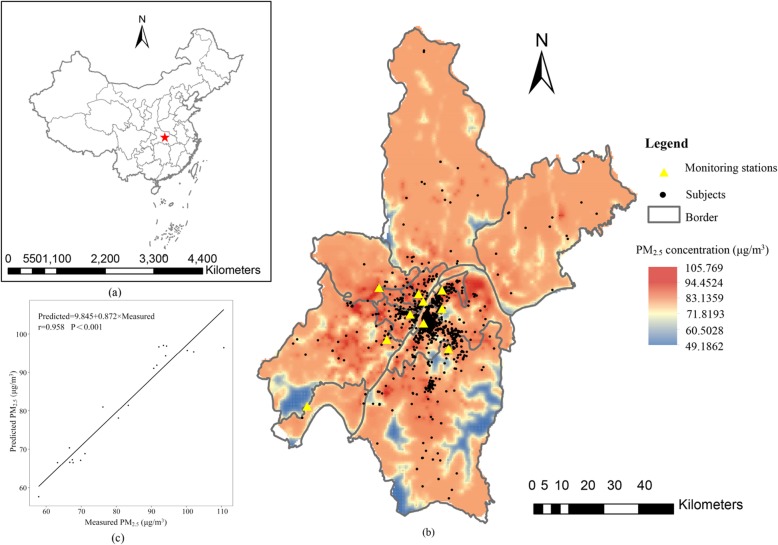
Geographical location of Wuhan in China (**a**). Spatial distribution of mean PM_2.5_ estimations across Wuhan city from January 1, 2013 to August 31, 2015 (**b**). A scatter plot correlating the measured and predicted PM_2.5_ values from 10 monitoring stations (**c**)

There are 10 national monitoring stations across Wuhan city, and the locations are shown in Fig. 2b. In this study, the daily air quality index (AQI) data of PM_2.5_ from 10 national monitoring stations were obtained from the Wuhan Environmental Protection Bureau website (http://www.whepb.gov.cn/) from January 1, 2013 to August 31, 2015. These monitors automatically and continuously, 24 h a day and 365 days a year, collect the concentration of specific air pollutants. We transformed the AQI data to pollutant concentration data according to the technical regulation on ambient AQI [23].

### Exposure assessment

The spatiotemporal exposure assessment in this study was based on a LUR model with 1 km spatial resolution. In the modeling process, we considered geographic predictor variables, including types of land use (https://earthexplorer.usgs.gov/), the length of roads (http://www.openstreetmap.org), the nearest distance between the station and the road, the number of industrial sources (http://www.whepb.gov.cn/), population density (https://sedac.ciesin.columbia.edu/), and digital elevation (http://srtm.csi.cgiar.org). In the study, it is unavailable for the birth data from June 1, 2014 and December 31, 2014. Given that the discontinuity of the birth data, two LUR models were built. In addition, compared with one LUR model, these two LUR models showed good performance of model fitness. LUR-model 1 was built to assess exposure for women who delivery during the first trimester (between November 1, 2013 and May 31, 2014). LUR-model 2 was built to assess exposure for women who delivery during the second period (between January 1, 2015 and August 31, 2015). The average PM_2.5_ concentration of each time period and the potential predictor variables derived from ArcGIS (ArcGIS 10.3) from all 10 monitors were used to develop the LUR models. In this study, LUR models performed well and yielded high leave-one-out-cross-validation (LOOCV) R^2^ value, which reached 0.808 and 0.910 for LUR-model 1 and LUR-model 2, respectively. The correlation coefficient between measured and predicted values from the two LUR models was 0.958 (Fig. 2c). Based on the estimated average concentration of PM_2.5_ during the two periods, we generated the average surface PM_2.5_ concentration from January 1, 2013 to August 31, 2015 across Wuhan city (Fig. 2b).

The Kriging interpolation method was used to transform predicted PM_2.5_ data from monitors into concentration maps. We extrapolated the average concentrations of PM_2.5_ for each time period to a daily level, following the method described in previous studies [22, 24]. First, we geocoded maternal addresses and assigned the period-specific average PM_2.5_ concentration from the LUR models to each woman. Second, daily PM_2.5_ concentration for each subject was adjusted by the ratio of daily-specific PM_2.5_ concentrations to the estimated period-specific average PM_2.5_ concentration at the nearest monitor. Finally, the average concentration of PM_2.5_ was assigned to each subject in accordance with four exposure periods—the entire pregnancy, first trimester, second trimester, and third trimester.

### Statistical analysis

The concentration of PM_2.5_ exposure was regarded as both a continuous and categorical variable in our analysis. We performed a logistic regression model to examine the association between PTB and PM_2.5_ exposure during the entire pregnancy and each trimester. First, we built a crude model with only PM_2.5_ concentration as a continuous independent variable. Then, we added maternal age (≤24, 25–29, 30–34, ≥35 years of age), years of education (≤9, 10–13, ≥14 years), delivery mode (vaginal or cesarean), gravidity (1 pregnancy or > 1 pregnancies), parity (delivering their first-born or mother with a previous live birth), gestational diabetes (yes or no), gestational hypertension (yes or no), season of conception [warm (March–August) or cold (September–February)] [17], and sex of infant (male or female) to build the adjusted models. In addition to analyzing PTB as a single outcome, we examined the birth outcome separately by subtypes, including MLPTB, VPTB, and ExPTB. As there was a small sample size of ExPTB (< 28 completed weeks, *n* = 2) in our sample, we did not include these births in the analyses. In these models, the results were showed by odd ratio (*ORs*) and its 95% confidence intervals (*CIs*) related to per 10 μg/m^3^ increase of PM_2.5_.

According to the quartiles of the distribution of PM_2.5_ concentration, we compared subjects in each higher exposure quartile with those in the first quartile (first quartile: <25th percentile; second quartile: 25th to 50th percentile; third quartile: 50th to 75th percentile; fourth quartile: >75th percentile) during the entire pregnancy and in each trimester. We examined whether specific subgroups were more vulnerable to the effect of maternal PM_2.5_ exposure, subgroups were stratified by sex of infant (male or female), years of education (≤9, 10–13, ≥14 years), and season of conception [warm (March–August) or cold (September–February)]. Stratified analyses were adjusted for maternal age, years of education, delivery mode, gravidity, parity, gestational diabetes, gestational hypertension, season of conception, and sex of infant, without each categorized variable. All above statistical tests were two-sided, and a *p-*value < 0.05 was considered statistically significant. All of the analyses were conducted using R 3.4.2 software (R Core Team, 2018).

## Results

### Characteristics of the participates

After exclusion, a total of 2101 deliveries were included in our study. Out of the 2101 deliveries, 273 were PTB, and the prevalence rate of PTB in this study was 13%. The average age of the pregnant women in this study was 30 years old. We found that there were appreciable differences in prevalence of PTB by maternal age, years of education, delivery mode, gravidity, parity, gestational hypertension and gestational diabetes. Among the PTB group, the percentages of women who were younger than 24 years old, had less than 9 years of education, were pregnant before, had gestational hypertension and gestational diabetes, and who conceived during the cold season were significantly higher than term birth group (Table 1).

**Table 1:** Characteristics of the study population, by term and preterm births (Wuhan, China, November 1, 2013 to May 31, 2014; January 1, 2015 to August 31, 2015)

Covariates	Preterm births		Term births		*p* value ^a^
	n	%	n	%	
Maternal age, years					0.018
≤ 24	24	18.6	105	81.4	
25–29	85	10.5	721	89.5	
30–34	73	13.1	486	86.9	
≥ 35	91	15.0	516	85.0	
Years of education					< 0.001
≤ 9	69	24.8	209	75.2	
10–13	62	17.5	293	82.5	
≥ 14	142	9.7	1326	90.3	
Sex of infant					0.335
Male	151	13.7	954	86.3	
Female	122	12.2	874	87.8	
Delivery mode					0.030
Vaginal	97	11.1	776	88.9	
Cesarean	176	14.3	1050	85.7	
Gravidity					
1	119	10.0	1076	90.0	< 0.001
≥ 2	154	17.0	752	83.0	
Parity					< 0.001
1	183	10.8	1504	89.2	
≥ 2	90	21.8	322	78.2	
Gestational hypertension					< 0.001
Yes	39	37.1	66	62.9	
No	234	11.7	1762	88.3	
Gestational diabetes					0.019
Yes	18	21.4	66	78.6	
No	255	12.6	1762	87.4	
Season conceived					0.045
Warm	169	12.0	1243	88.0	
Cold	104	15.1	585	84.9	

^a^*p* value for Chi-square test for categorical variables

### Spatial distribution of PM_2.5_

The spatial distribution of the PM_2.5_ concentration in this study period across Wuhan is presented in Fig. 2b, with a spatial resolution of 1 km × 1 km. The average concentration of PM_2.5_ during this study period was 81.30 μg/m^3^ in Wuhan. The concentration of PM_2.5_ was high within the main urban areas while it was low within the new urban areas (Fig. 2b). Most of the participants lived within the main urban area. Monitors were located on areas with a relatively high population density; therefore, the modeled values would be able to adequately represent the population exposure (Fig. 2b).

**Fig. 3:**
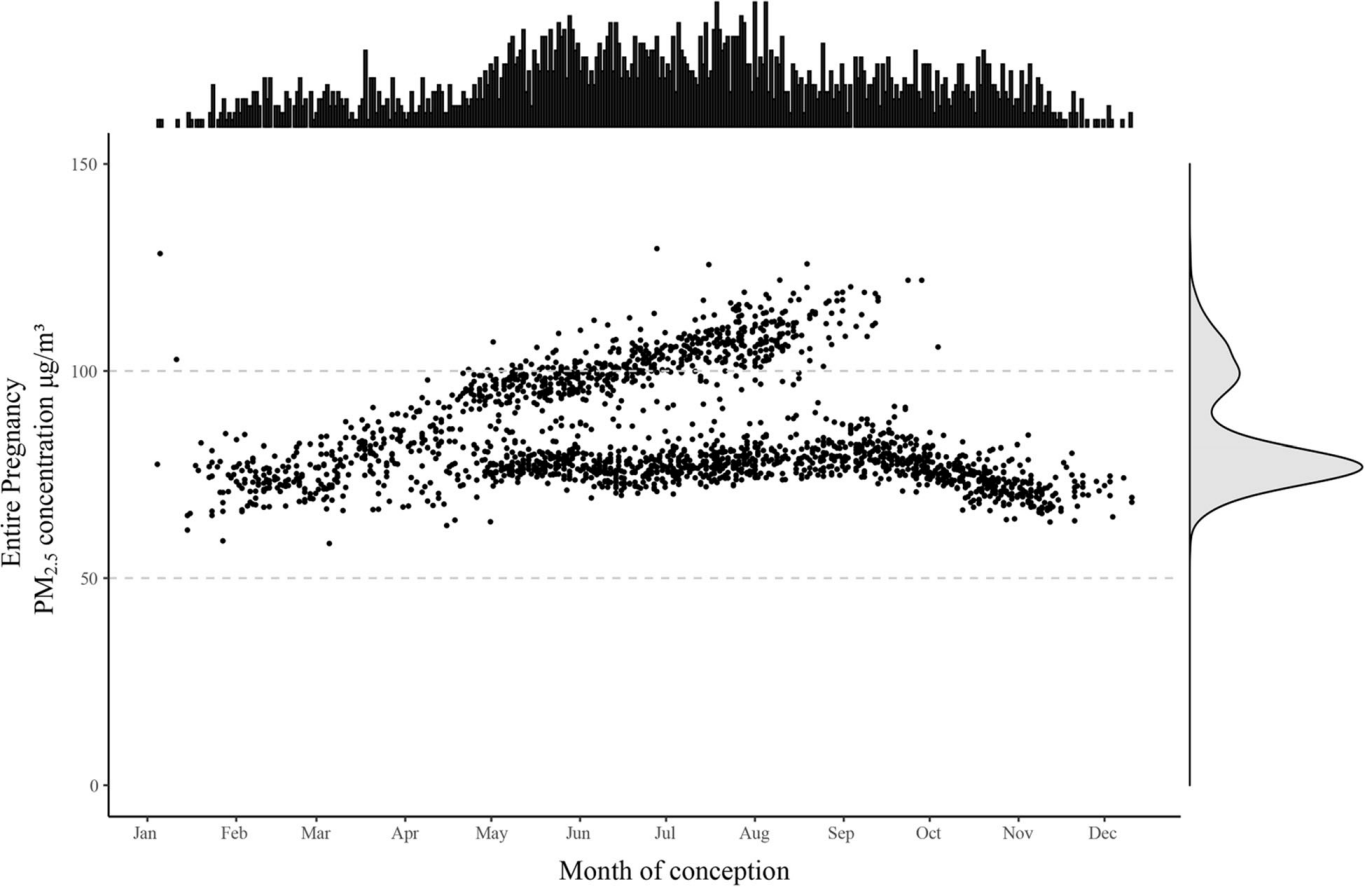
Distribution of months of conception for participants and individual PM_2.5_ exposure in the specific trimester. Notes: The height of the bar indicates the number of participants. The dot indicates the woman who exposure to a specific PM_2.5_ concentration. The density plot on the right marginal of y-axis visualizes the distribution of the number of subjects over the PM_2.5_ concentration intervals

During the whole pregnancy period, the average concentration of PM_2.5_ exposure was 84.54 μg/m^3^, and the exposure level ranged from 58.53 μg/m^3^ to 129.53 μg/m^3^. Since there were two live births prior to 28 completed weeks of gestation, they did not have exposure in the third trimester. The exposure level of PM_2.5_ for pregnant women in the first trimester (69.12 μg/m^3^) was lower than the exposure level in the second trimester (92.28 μg/m^3^) and third trimester (92.22 μg/m^3^; Table 2). The distribution of subjects’ average PM_2.5_ exposure during the whole pregnancy was shown in Fig. 3. In addition, months of conception for most of participants were distributed between May and September.

**Table 2:** Average exposure level of PM_2.5_ in each time window based on LUR model estimates of the study population in Wuhan, China

Exposure	N	Mean	Min	Percentiles of exposure			Max
				P25	P50	P75	
Entire pregnancy	2101	84.54	58.53	75.39	79.27	95.49	129.53
Trimester 1	2101	69.12	30.49	50.20	65.59	86.60	147.83
Trimester 2	2099	92.28	33.03	68.71	89.80	107.69	188.16
Trimester 3	2099	92.22	21.90	61.05	87.96	114.94	207.17

*Abbreviations:* Min minimum, *P25* 25th percentile, *P50* 50th percentile, *P75* 75th percentile, *Max* maximum

### Association between PM_2.5_ exposure and PTB

Compared with the results from the crude model, the *OR*s for PTB, MPTB, and VPTB became larger during each exposure window when the potential confounders were adjusted in the model. Except for the third trimester, the *OR*s for VPTB in other exposure windows were larger than MPTB. For PTB and subtypes of PTB, the highest *OR*s all appeared in the first trimester (*OR* and 95% *CI*: 1.169 [1.077, 1.262], 1.170 [1.071, 1.269], 1.265 [1.116, 1.417] for PTB, MPTB, and VPTB, respectively) (Table 3).

**Table 3:** Odd Ratios and 95% *CI*s of categorial PTB for per 10 μg/m^3^ increase in PM_2.5_ during each time window based on LUR model estimates

	N	Crude model		Adjusted model ^a^	
		*OR* (95% *CI*) value	*p* value	*OR* (95% *CI*) value	*p* value
PTB	273				
Entire pregnancy		1.191 (1.097, 1.285)	< 0.001	1.263 (1.158, 1.368)	< 0.001
First trimester		1.127 (1.069, 1.185)	< 0.001	1.169 (1.077, 1.262)	< 0.001
Second trimester		1.039 (1.001, 1.077)	0.040	1.056 (1.015, 1.097)	0.007
Third trimester		1.001 (0.967, 1.036)	0.937	1.052 (1.002, 1.101)	0.041
MPTB	230				
Entire pregnancy		1.183 (1.081, 1.286)	< 0.001	1.230 (1.118, 1.344)	< 0.001
First trimester		1.107 (1.044, 1.171)	0.001	1.170 (1.071, 1.269)	0.001
Second trimester		1.040 (1.000, 1.080)	0.052	1.051 (1.008, 1.094)	0.021
Third trimester		1.014 (0.977, 1.052)	0.460	1.053 (1.000, 1.106)	0.048
VPTB	41				
Entire pregnancy		1.192 (0.963, 1.426)	0.101	1.496 (1.222, 1.778)	< 0.001
First trimester		1.203 (0.963, 1.449)	0.098	1.265 (1.116, 1.417)	< 0.001
Second trimester		1.036 (0.946, 1.127)	0.431	1.111 (1.005, 1.218)	0.040
Third trimester		0.924 (0.833, 1.157)	0.104	1.054 (0.925, 1.186)	0.413

*Abbreviations: OR* odds ratio, *CI* confidence interval, MPTB moderate preterm births (32–37 weeks), VPTB very preterm births (28–32 weeks)

^a^ Logistic regression model, adjusted for maternal age, years of education, delivery mode, gravidity, parity, season of conception, gestational diabetes, gestational hypertension, and sex of infant

**Fig. 4:**
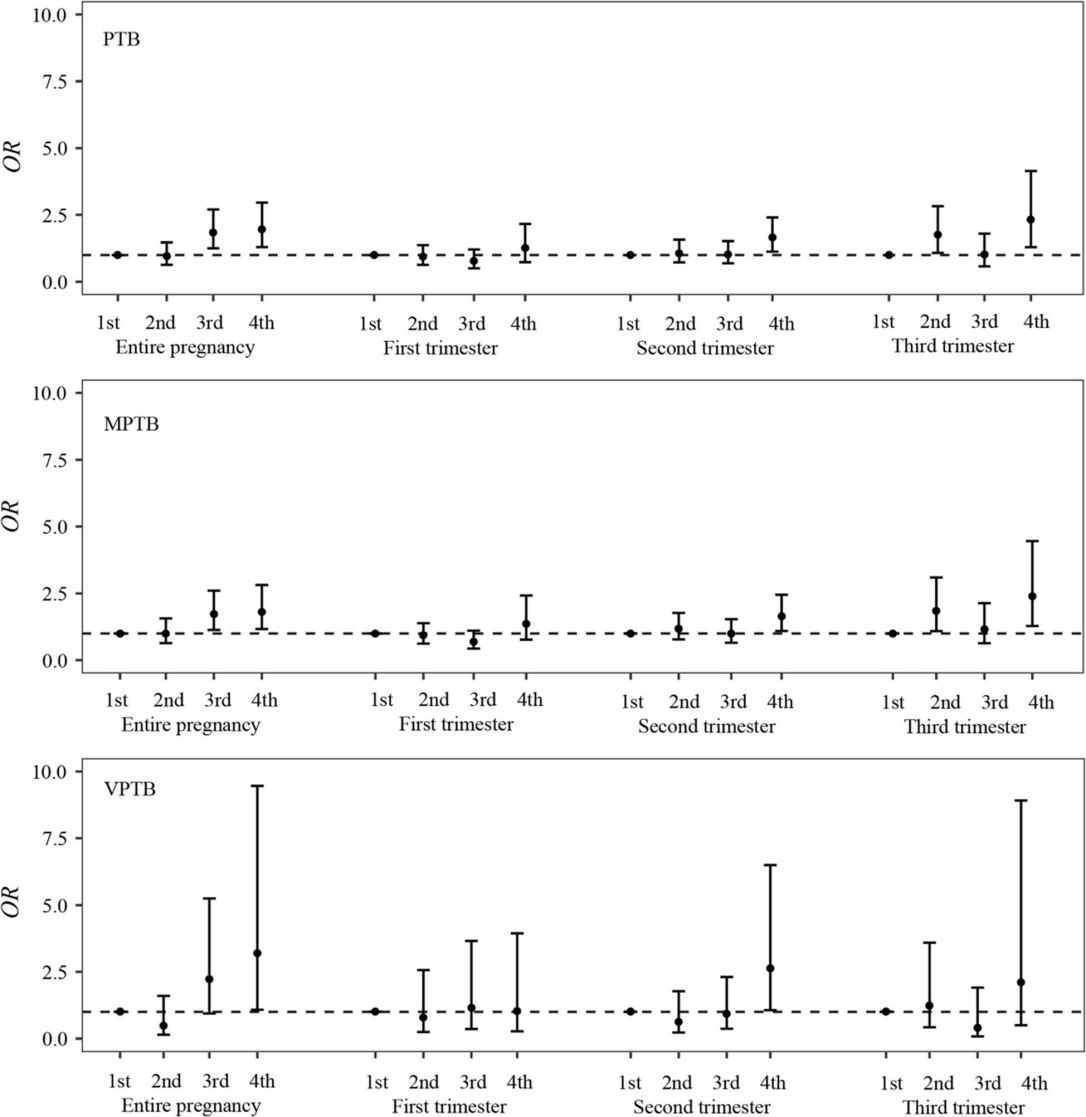
Adjusted odd ratios (95% *CI*s) of VPTB, MPTB, and PTB by entire pregnancy and trimester-specific exposure quartile. Logistic regression models were adjusted for maternal age, years of schooling, delivery mode, gravidity, parity, season of conception, gestational diabetes, gestational hypertension, and sex of baby. Abbreviations: *OR*, odds ratio; PTB, preterm births (<37 weeks); MPTB, moderate preterm births (32–37 weeks); VPTB, very preterm births (28–32 weeks); 1st, the first quartile; 2nd, the second quartile; 3rd, the third quartile; 4th, the fourth quartile

After adjustment of covariates, the risk of PTB, MPTB, and VPTB increased with quartiles of PM_2.5_ exposure during the entire pregnancy period. We observed a 219.4% (*OR*: 3.194; 95% *CI*: 1.078, 9.461), 81.4% (*OR*: 1.814; 95% *CI*: 1.168, 2.817), and 96.1% (*OR*: 1.961; 95% *CI*: 1.300, 2.957) increase in the risk of VPTB, MPTB, and PTB for women in the highest PM_2.5_ entire-pregnancy exposure quartile, respectively. Except for the first trimester, the effects of PM_2.5_ exposure on PTB and MPTB in the highest quartile during the two trimesters were statistically significant. For VPTB, although compared with the first quartile of PM_2.5_ exposure, the higher quartiles of PM_2.5_ exposure in each trimester not significantly associated with the risk of VPTB. A trend was found towards an increased risk of VPTB with increased quartile of PM_2.5_ exposure (Fig. 4.).

The stratified analysis showed that the association between PM_2.5_ and PTB varied by maternal education, infant sex, and season conceived (Table 4). The effect of PM_2.5_ during the entire pregnancy appeared to be stronger on women who received less than 9 years of education. For the entire pregnancy, there was no difference between women who delivered a male infant (*OR*: 1.257; 95% *CI*: 1.107, 1.409) and those who delivered a female infant (*OR*: 1.250; 95% *CI*: 1.104, 1.398) in the health effect of PM_2.5_ exposure. Except for the third trimester, women who conceived during the cold season were more sensitive to PM_2.5_ exposure than those that conceived during the warm season. Women who conceived during the warm season were more susceptible to PM_2.5_ exposure during the third trimester than those that conceived during the cold season.

**Table 4:** Adjusted odd ratios of preterm births for each 10 μg/m^3^ increment in PM_2.5_ exposure during the entire pregnancy and trimesters in each subtype

Subgroup	N	Entire pregnancy		First trimester		Second trimester		Third trimester	
		*OR* (95% *CI*)^a^	*p* value	*OR* (95% *CI*)^a^	*p* value	*OR* (95% *CI*)^a^	*p* value	*OR* (95% *CI*)^a^	*p* value
Maternal education, years									
≤ 9	278	1.327 (1.115, 1.542)	0.002	1.154 (0.954, 1.359)	0.132	1.125 (1.039, 1.211)	0.004	1.060 (0.945, 1.172)	0.292
10–13	355	1.148 (0.905, 1.397)	0.235	0.991 (0.792, 1.193)	0.927	1.034 (0.946, 1.123)	0.447	1.097 (0.986, 1.210)	0.088
≥ 14	1468	1.282 (1.139, 1.427)	< 0.001	1.247 (1.123, 1.374)	< 0.001	1.035 (0.979, 1.092)	< 0.001	1.036 (0.970, 1.103)	0.287
Infant sex									
Female	997	1.250 (1.104, 1.398)	< 0.001	1.110 (0.981, 1.240)	0.095	1.054 (1.000, 1.110)	0.058	1.076 (1.006, 1.147)	0.033
Male	1104	1.257 (1.107, 1.409)	< 0.001	1.233 (1.100, 1.370)	< 0.001	1.050 (0.990, 1.110)	0.106	1.033 (0.962, 1.105)	0.360
Season of conception									
Warm	1412	0.998 (0.873, 1.126)	0.978	0.968 (0.853, 1.083)	0.584	1.026 (0.980, 1.072)	0.258	1.086 (1.031, 1.140)	0.002
Cold	689	1.882 (1.656, 2.114)	< 0.001	1.637 (1.442, 1.835)	< 0.001	1.175 (1.083, 1.267)	< 0.001	0.838 (0.697, 0.981)	0.027

*Abbreviation: OR* odds ratio, *CI* confidence interval

^a^ Adjusted for maternal age, years of education, delivery mode, gravidity, parity, season of conception, gestational diabetes, gestational hypertension, and sex of infant, without each categorized variable

## Discussion

Our study adds to the growing body of evidence that demonstrates that the risk of premature birth increases with PM_2.5_ exposure during pregnancy. Maternal PM_2.5_ exposure was more strongly associated with VPTB than MPTB. For VPTB and MPTB, PM_2.5_ exposure during different time windows had a different effect on them. These two subgroups of PTB had a different sensitivity to the increase in PM_2.5_ exposure level.

In recent years, there has been increasing evidence that maternal PM_2.5_ exposure increases the risk of PTB [12, 25]. The results of most studies were consistent and indicated that PM_2.5_ exposure during the entire pregnancy was associated with PTB, with the *OR*s ranging from 1.01 to 1.87 for per 10 μg/m^3^ increment in PM_2.5_ [25–28]. Previous meta-analyses also found estimated *OR* for PTB each 10 μg/m^3^ increment in PM_2.5_ exposure during the entire pregnancy being 1.13 (95% *CI*: 1.03, 1.24) [19]. Compared with those previous studies, our study observed a higher magnitude association between PM_2.5_ exposure and PTB (*OR*: 1.263; 95% *CI*: 1.158, 1.368). The difference between our findings and those of other studies might be due to the following possible reasons: First, the information for mother-infant pairs in this study was collected from a single hospital, and women with a poor physical condition were more likely to deliver in this hospital. Thus, the specificity of the study population could lead to overestimate the hazard of PM_2.5_ exposure on PTB. Second, the variability of exposure assessment could be another possible reason. Many studies used fixed-site measurements as individual exposure, while our study used high-resolution LUR model predictions. Third, the disparity in the proportions of PTB with different gestational ages (MPTB, VPTB, and ExPTB) among our study and previous studies also affected the estimation of the association.

In the subgroup analyses of PTB, we found that the hazard of PM_2.5_ exposure during all exposure windows, except the third trimester, on VPTB was stronger than MPTB. Furthermore, the effects of PM_2.5_ exposure in the first trimester on VPTB and MPTB appeared to be stronger than in other two trimesters. These results were consistent with those of previous studies. One study in California found that exposure to local traffic-generated pollutants (e.g., PM_2.5_, NO_x_) increased the risk of premature birth, and the risk of PM_2.5_ exposure was stronger for VPTB than MPTB [16]. Previous studies in Hong Kong and the Chinese mainland reported that the impact of PM_2.5_ exposure during pregnancy was more significant on PTB with a younger gestational age than PTB with an older gestational age, and the most susceptible exposure window was the first trimester [19, 29]. One potential explanation is that the first trimester is the critical stage for embryo implantation and placenta formation [30], and the production of free radicals induced by air pollution might cause an inflammatory response, increasing blood viscosity [30, 31]. Suboptimal placenta perfusion from blood viscosity changes may cause adverse pregnancy outcomes, including low birth weight and PTB. PM_2.5_-associated inflammation could artificially cause the placenta to age prematurely and may partially explain why we observed that VPTB had the greatest risk [32]. This finding was important because postnatal health impairments were greatest for children born extremely premature [33]. Infants born at the youngest gestational age not only had a greater risk of mortality and morbidity but also had a greater risk of long-term complications, such as hypertension, respiratory impairment, and immunologic impairment [34, 35].

The exposure-response relationship has been a critical issue in characterizing the health impacts induced by air pollutants. Previous studies have observed that the risk of PTB and categorical PTB (MPTB, VPTB) increased with quartiles of PM_2.5_ exposure during the entire pregnancy. This previous finding was consistent with the results of our study [16]. However, for the exposure-response relationship between PM_2.5_ exposure and premature birth, previous studies either failed to consider exposures in each specific trimester or did not divide PTB into various subtypes. In this study, we observed that compared with the first quartile of PM_2.5_ exposure, higher quartiles of PM_2.5_ exposure during any trimesters did not significantly increase the risk of VPTB. For MPTB, we found that women exposed at the fourth quartile of PM_2.5_ experienced a higher risk compared with those exposed at the first quartile. This result suggested that the exposure-response relationship of these two types of premature birth may be different at a relatively high exposure level (81.30 μg/m^3^). At a relatively high exposure level, the effect of PM_2.5_ exposure on VPTB reached its maximum at the second quartile level. However, the risk of MPTB continued to augment as the level of PM_2.5_ exposure increased. Additionally, considering the sample size of VPTB, we were not able to accurately observe the dose-response relationship between high levels of PM_2.5_ exposure and VPTB. In any case, this is an isolated finding with no precedent in the literature, and therefore it should be regarded with caution.

Consistent with other studies, this study found that the hazard of PM_2.5_ exposure during the entire pregnancy on PTB was stronger for women who conceived during the cold season than who conceived during the warm season. To our knowledge, higher temperature facilitates cyclones, which is advantageous for the diffusion of pollutants. Compared with women who conceived during the warm season, women who conceived during the cold season experienced a longer period with high pollution, and early pregnancy is a critical period for the formation of the placenta, which appears to be more susceptible to PM_2.5_ exposure [30]. In addition, cold temperature could lead to elevated blood viscosity and vascular constriction and increased exposure to risk factors of PTB [36], such as air pollution and passive smoking [37]. We also found that most of women conceived during the warm season, which may be because of the education policy regarding the school age of children in China. Most mothers want their children to be born before September in order to make them eligible for admission to school in the earliest month of September possible. Our study found no remarkable difference in the PM_2.5_-PTB association between women with male infants and women with female infants. We also observed that women who had the lowest education had a higher risk of PTB associated with PM_2.5_ exposure during their entire pregnancy, which was consistent with the results of previous studies. Possible reasons for these differences in associations may include differences in the amount of time spent outdoors, knowledge of prenatal care, and baseline health status for pregnancy women [38, 39].

This study had several strengths: First, we used a LUR model and Kriging interpolation for individual exposure assessment. This approach uses known samples to predict air pollution concentrations at unknown locations after considering for other geographic factors. Consequently, these estimated PM_2.5_ data have an increased spatial and temporal resolution compared to the measurement from fixed-monitoring station. Second, we examined the association between maternal PM_2.5_ exposure and various subtypes of PTB. Dividing PTB into three categories may help reveal association differences, which have been masked by folding all categories of PTB into a single outcome.

Limitations should be considered when extrapolating the study outcomes. Firstly, the information of mother-infant pairs in this study was collected from a single hospital. The hospital in this study is one of the best hospitals in Wuhan with advanced medical technology and better health care. Women in poor physical conditions prefer to deliver at this hospital; thus, there might be some selection bias, which may limit the generalization of the findings. Secondly, limited by the overall population in our study, the number of VPTB is relatively small. However, we found that PM_2.5_ exposure during pregnancy had a larger effect on VPTB than MPTB, although VPTB group had a wider 95%*CI* than MPTB and PTB. Thirdly, the study was lack of health baseline information (vitro fertilization, a prior experience of cesarean section, and maternal obesity, etc.) and behavioral information (smoking status, alcohol consumption, etc.) for pregnancy women. Although we could not obtain information on smoking status and alcohol consumption during pregnancy, phenomenon of smoking and alcohol consumption are rare among Chinese pregnancy women due to the influence of traditional Chinese culture [40]. Therefore, we believed that these confounders have less influence on the results. Fourthly, individual exposure was assessed based on the address in the medical records. Owing to the restriction of medical data, we can not obtain other individual exposure information, such as residential mobility, frequency of outdoor activities and availability of a purification machine, which might be important for assessing individual exposure. However, one study reported that the mobility rate of pregnant Chinese women was relatively low in Wuhan, China (8.4%) [41, 42]. Thus, we concluded that the influence of maternal mobility on the results in our study was limited.

## Conclusion

In conclusion, the results of this study suggest that the exposure to ambient PM_2.5_ during pregnancy increases the risk of premature birth. The risk might vary by different subtypes of PTB and by different exposure windows. The effects of PM_2.5_ exposure on PTB appear to be stronger in women who receive less years of education or conceive during a cold season. Findings from this study could be important for policy makers in applying research evidence to policy, such as inclusion of the estimated effects in the future revisions of air quality standards. Also, public policies should be developed to prevent pregnant women from the impact of air pollution, especially among those vulnerable pregnant women.

## Data Availability

The dataset used and analyzed during the current study is available from the corresponding author on reasonable request.

## Abbreviations

LUR: Land Use Regression
PTB: Preterm birth
MPTB: Moderate preterm birth
VPTB: Very preterm birth
ExPTB: Extremely preterm birth
